# Development of a Tool for Measuring Ventilator-Associated Pneumonia Prevention Behaviors of Intensive Care Unit Nurses

**DOI:** 10.3390/ijerph19148822

**Published:** 2022-07-20

**Authors:** Sungjung Kwak, Sujeong Han

**Affiliations:** 1 Robotic Surgery Center, Konyang University Hospital, Daejeon 35365, Korea; sungjunglob@hanmail.net; 2 College of Nursing, Konyang University, Daejeon 35365, Korea

**Keywords:** ventilator-associated, pneumonia, intensive care unit, nurse, mechanical ventilation

## Abstract

**Introduction:** Ventilator-associated pneumonia (VAP) lengthens intensive care unit (ICU) stays and increases medical expenses and mortality risk. **Aim:** We aimed to develop and validate a tool for measuring VAP prevention behaviors among ICU nurses. **Method:** This is a methodological study that included a tool development step, based on the procedure suggested by DeVellis, and a tool verification step. **Results:** Through a literature review and focus interviews, 35 preliminary items were selected. After a content validity examination by experts and a pre-test, 30 items were chosen for this study. In the testing phase of the main survey, the final version tool was used on 452 ICU nurses to assess validity and reliability. From factor analysis, 7 factors and 17 items were selected. The factors included aspiration prevention, ventilator management, oral care, suction system management, subglottic suction, spontaneous awakening trials and spontaneous breathing trials, and standard precautions. The total determination coefficient was 71.6%. These factors were verified using convergent, discriminant, and concurrent validity tests. Internal consistency reliability was acceptable (Cronbach’s α = 0.80); thus, the VAP prevention behavior measurement tool was proven valid and reliable. **Conclusions:** This tool can be used with ICU nurses to measure behaviors associated with VAP prevention and, in turn, VAP prevention measures can be improved.

## 1. Introduction

Ventilator-associated pneumonia (VAP) is the most common infectious disease in patients requiring mechanical ventilation in the intensive care unit (ICU) [1]. VAP occurs when a patient who does not have symptoms of pneumonia or an infection during the incubation period develops the disease from at least 48 h of mechanical ventilation and intubation, up to another 48 h after removal [2]. Once a patient develops VAP, they are hospitalized in the ICU for an extra 4–9 days, with a mortality rate twice as high as in individuals without VAP [3]. VAP reportedly causes 36,000 deaths each year in the United States [4]. There are approximately 18,900 confirmed cases of VAP in Europe annually, with reported mortality rates of 50–76% for serious cases [4]. In Korea, 52.6% of all patients with pneumonia had VAP, with a mortality rate of 24–50%. Of the patients, 70% were in the high-risk group [5]; thus, many patients on mechanical ventilation in ICUs are developing VAP, which lengthens the hospitalization period and increases medical expenses and the risk of mortality. Due to its effects on patients, families, and society, VAP is a medical issue that must be prevented.

To prevent VAP, the “bundle approach” idea emerged, which refers to a group of preventative interventions. The Institute of Healthcare Improvement (IHI) in the United States announced a VAP prevention bundle including four elements: elevation of the head of the bed, peptic ulcer disease (PUD) prophylaxis, deep venous thrombosis (DVT) prophylaxis, and daily sedative interruption and assessment of readiness to extubate [6]. European countries also announced guidelines to be used in practice, which consist of five categories that consider the priority and significance among VAP prevention interventions: non-ventilator circuit changes unless specifically indicated, alcohol hand hygiene, appropriately educated and trained staff, incorporation of sedation control and weaning protocols into patient care, and oral care with chlorhexidine [7]. The Korea Disease Control and Prevention Agency (KDCA) also announced a VAP prevention bundle consisting of five categories that refer to the assessment standards of domestic medical institutions based on the guidelines from the Centers of Disease Control and Prevention (CDC): elevation of the head of the bed, daily sedative interruption and assessment of readiness to extubate, peptic ulcer disease (PUD) prophylaxis, deep venous thrombosis (DVT) prophylaxis, and provision of daily oral care using chlorhexidine [8]. Research on the prevention of VAP has followed, and the preventative effects of various interventions have been proven through studies on the prevalence of VAP. The studies considered factors such as ventilator circuit replacement cycle [9], oral care [10], suction [11], subglottic secretion drainage and position [12], endotracheal tube cuff pressure [13,14], and tracheostomy tube management [15]. Consequently, preventative activities proven through various research studies are being practiced in ICUs with VAP prevention bundles [16].

Institutions across the world have announced VAP prevention bundles and conducted various studies to identify preventative actions; however, the actual exercise of VAP prevention activities in clinical practice is unclear. This is found to be highly related to the prevalence rate of the disease [17]. Effective interventions are not offered to patients every day, given the busy hours in ICUs, which is the same situation for VAP prevention [18]. European researchers studied the implementation of the prevention bundle in hospitals and reported that all five categories of the bundle had low compliance rates [19]. Although guidelines have been recommended to provide consistent care and reduce variability in the clinical setting, perfect implementation of the guidelines is not guaranteed and depends on many factors [20]. One of the strategies essential to the successful implementation of these guidelines is performance measurement and feedback [21]. Concanour et al. [22] found that the VAP prevention bundle alone did not decrease the incidence of VAP; rather, they found that the VAP incidence rate was significantly reduced after the implementation was measured daily and feedback was provided. Moreover, Hawe et al. [18] reported that the VAP prevention bundle compliance rate improved after the implementation of multiple programs, including a VAP prevention bundle compliance measurement. The VAP incidence rate decreased significantly from 19.2 to 7.5 per 1000 ventilator days. To evaluate the effectiveness of such prevention strategies, valid and reliable data monitoring (measurement of implementation) is required, which has long been identified as an effective method of reducing infection [23].

Of the tools developed overseas for measuring the implementation of VAP preventive behaviors, the use of an Evac tube capable of subglottic suction [23], daily replacement of heat and moisture exchangers, provision of analgesic prior to suction [24], and education for the patient’s family on oral care or passive joint movement in the ICU and their encouragement to participate [25] are not prevalent of ICUs in Korea. In addition, only 50% of the total items were related to VAP prevention, and the large number of items (76) [26] made it difficult for nurses to measure them daily in busy ICUs.

In Korea, Ban’s tool [27], developed in 2007 based on the CDC guidelines, is mainly used; however, the revised VAP prevention guidelines announced by the Korea Disease Control and Prevention Agency (KDCA) in 2017 changed the current clinical field in the intensive care unit and Ban’s tool was not reflected. In addition, intensive care nurses needed to evaluate a total of 43 items daily, which was found to be too many for them; there are limitations because the items were developed only through content validity and reliability measured by experts. Most of the tools [28,29,30,31] developed after Ban’s tool [27] only added revisions and supplements to Ban’s tool [27] or only evaluated content validity or reliability after revisions. In addition, almost no tools have been developed according to the systematic methodological procedure of tool development.

While the effects of VAP prevention bundles are valid across the world, the components of the bundles in each country and institution are different. Therefore, it is necessary to integrate commonly recommended behaviors. In addition, many interventions, other than the VAP prevention bundle, that have been proven through studies are being implemented in ICUs; however, they are not being managed properly. VAP has various causes; therefore, there is a need for a measurement tool that can comprehensively integrate preventive interventions that are not included in the VAP prevention bundle so that nurses in ICUs in Korea can easily adopt them in practice. This study aimed to define VAP prevention behaviors for ICU nurses by applying a hybrid model and verifying its validity and reliability by developing a measurement tool [31]. In addition, this study sought to provide basic data for the development and study of tools to accurately evaluate the implementation of VAP prevention behaviors and increase the implementation rate.

### Aim

This study aimed to develop a tool that measures the VAP prevention behaviors of ICU nurses and verify its validity and reliability.

## 2. Materials and Methods

### 2.1. Study Design

In this study, we developed a tool to measure the implementation of VAP prevention behaviors by intensive care unit nurses. This is a methodological study to verify validity and reliability.

### 2.2. Study Procedure

**Figure 1:**
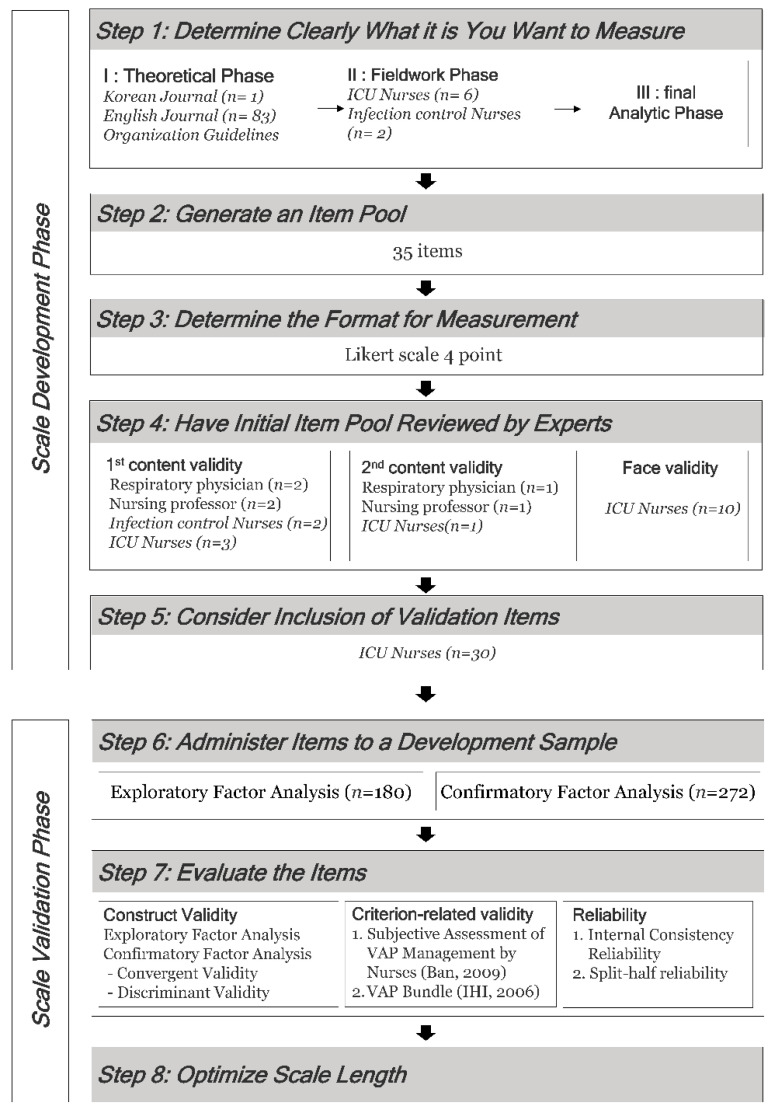
Research design and methods (Ban, 2009 [27], IHI, 2006 [6]).

The study included a tool development step, where items were developed based on the tool development and verification procedure suggested by DeVellis [32], and a tool verification step to check the tool’s validity (Figure 1).

#### 2.2.1. Phase 1: Scale Development Phase

Step 1. Determine What You Want to Measure Clearly

In this study, a hybrid model [31] was adopted to identify the concept of VAP preventive behaviors for nurses in the ICU. This is a useful method for investigating concepts used in the real field. It comprises three stages: theoretical, fieldwork, and final analytical phases.

(1) Theoretical Phase

**Figure 2:**
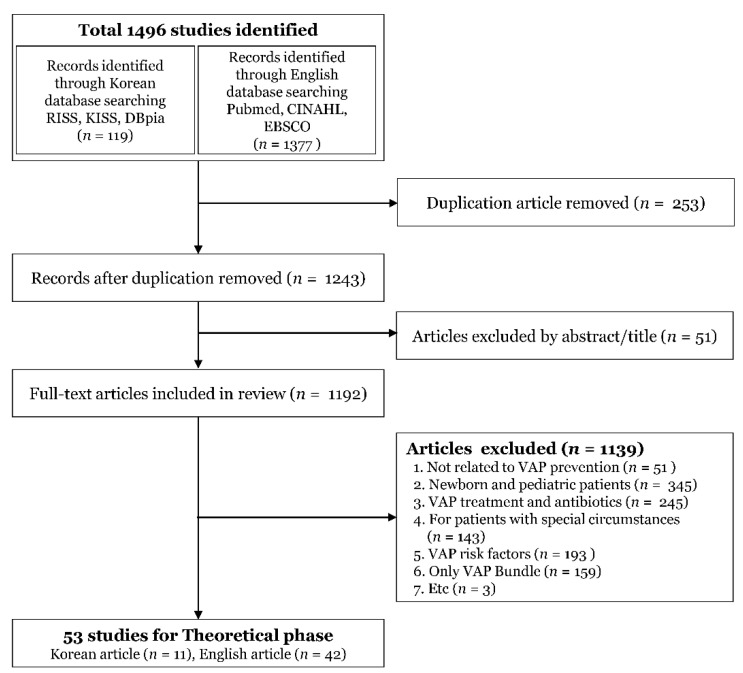
Literature search flow chart.

Previous studies conducted in and outside Korea were reviewed to derive the notional characteristics of VAP prevention behaviors and constituent factors. The major keywords found on RISS, DBpia, KISS, Pubmed, CINAHL, and EBSCO (the academic databases operated in Korea and abroad) were “ventilator associated pneumonia”, “ventilator-associated events”, “prevention”, “compliance”, and “adherence”, which were core concepts of this study. There were no restrictions on the year of publication for domestic literature; for foreign literature, academic journals and dissertations published after 2000 were searched (Figure 2).

With regard to domestic guidelines, the KDCA’s “Standard Guidelines for Prevention of Medical-related Infections” [8] were reviewed. Furthermore, the following overseas guidelines were reviewed: CDC, “Guidelines for preventing health-care-associated pneumonia” [33]; Society for Healthcare Epidemiology of America (SHEA), “Strategies to prevent ventilator-associated pneumonia in acute care hospitals: 2014 update” [34]; Association of Medical Microbiology and Infectious Disease (AMMI), “Clinical practice guidelines for hospital-acquired pneumonia and ventilator-associated pneumonia in adults” [35]; Institute for Healthcare Improvement (IHI), “How-to-guide: prevent ventilator-associated pneumonia” [6]; Institute for Clinical Systems Improvement (ICSI), “Health care protocol: prevention of ventilator-associated pneumonia” [36]; American Hospital Association (AHA), “Preventing ventilator-associated events change package: 2018” [25].

(2) Fieldwork Phase

A focus group interview was conducted to clarify the concept in the practical field of implementation of VAP prevention behaviors. The factors and items suggested in the theoretical research stage and terms used in the field were confirmed. The participants included six nurses in the ICU and two nurses working in the infection control room. After conducting one focus group interview, the insufficient parts were supplemented through six additional individual interviews with six nurses in the ICU. Conversations were voice recorded after receiving consent from the participants. The recorded interview contents were converted for documentation. Subsequently, through repeated reading, meaningful phrases, or sentences related to the implementation of VAP prevention, behaviors were selected and converted into a form for representation.

(3) Final Analytic Phase

A researcher and a professor from the nursing department reviewed the items by comparing and analyzing the results of the literature review in the theoretical stage with the data analyzed in the field study. Next, items with similar meanings or overlapping content were incorporated; 44 items were related to nine factors: aspiration prevention, oral care, subglottic suction, stress ulcer prophylaxis, deep vein thrombosis prophylaxis, spontaneous awakening and spontaneous breathing trials, ventilator circuit management, suction system management, and standard precaution.

Step 2. Generate an Item Pool

After repeatedly reviewing the 44 items derived from the concept identification stage through consultation with a researcher and a professor with experience in tool development from the nursing department, 35 preliminary items were finalized and nine items with similar meanings were deleted.

Step 3. Determine the Format for Measurement

The tool is composed of a 4-point Likert scale, excluding a neutral response, determining the degree of the participant’s attitude and accurately identifying their condition. The scales and points were “I never do it”, 1 point; “I seldom do it”, 2 points; “I mostly do it”, 3 points; and “I always do it”, 4 points.

Step 4. Have Initial Item Pool Reviewed by Experts

(1) Content Validity

Expert opinions were collected twice and the content validity of the preliminary items were tested. The nine experts consisted of two respiratory physicians, two nursing professors, two infection control nurses, and three ICU nurses.

To select valid items, the item content validity index (I-CVI), scale content validity index (S-CVI), and modified kappa values for preliminary items were calculated. As a result of the primary content validity, the S-CVI/average was 0.90; I-CVI score, 0.78 or less; and modified kappa, less than 0.75. A total of 5 items were excluded from these criteria: four items were excluded according to this criterion, and one item was excluded in expert opinion as a duplicate.

The second content validity evaluation was conducted 12 days after the first content validity evaluation. The expert panel consisted of one respiratory physician, one nursing professor, and one ICU nurse. The I-CVI and modified kappa value were 1.00 for all items, and the S-CVI/average was 1.00, which secured content validity for the 30 items. In addition, the adverb “must be” from the phrase “must be performed in a semi-recumbent position while supplying enteral feeding to reduce the occurrence of gastroesophageal reflux” was deleted. Following the experts’ opinions that the context needed to be revised, “the end of the ventilator” was edited to “the end of the ventilator circuit”, and “will be contaminated by secretions” to “clothes will be contaminated with secretions”.

(2) Face Validity

To check the face validity of the 30 items with confirmed content validity, opinions were collected from 10 ICU nurses on whether there were difficult-to-understand words, ambiguous sentences, or confusing items. In the I-CVI index, all items were 1.00, and explanations were added in response to the opinion that the terms “subglottic secretion” and “drip feed” were unfamiliar.

Step 5. Consider the Inclusion of Validation Items

The participants in the preliminary survey had the same conditions as those in this study, and those who participated in the face validity study were excluded. A total of 30 ICU nurses were sampled, including nine ICU nurses with 1–5 years of experience, nine ICU nurses with 5 or more but less than 10 years of experience, and 12 ICU nurses with more than 10 years of experience. It took nine minutes on average to complete the preliminary survey, and the Cronbach’s α value was 0.83.

#### 2.2.2. Phase 2: Scale Validation Phase

Step 6. Administer Items to a Development Sample

(1) Research Method

This survey consisted of a total of 83 items, including five items on general characteristics, 30 items on the tool developed by the researcher, 43 items on Ban’s [27] questionnaire on the “ventilator-associated pneumonia management survey” for criteria validation, and five items on IHI’s VAP bundle [8].

(2) Subjects and Data Collection Method

The participants in this survey were nurses working in the ICU of a general hospital with more than 800 beds. Those who voluntarily gave consent to participate in the study were included. The study excluded new nurses, those who were in the orientation period for transfer, and those in management (head nurse or chief nurse) who were not directly involved in patient care. The data collection for this survey was conducted from 20 November 2019 to 30 April 2020. The data of 452 patients were collected from two general hospitals with more than 800 beds and an online survey system using the same criteria.

Among the 452 respondents, 180 were extracted for exploratory factor analysis through random sample selection, and the remaining 272 were assigned for confirmatory factor analysis. The general characteristics of the two datasets showed no statistically significant differences. The general characteristics of the participants are detailed in Table 1.

This study was conducted after receiving approval from the Bioethics Committee of the university hospital to which the researcher belonged (IRB File No. KYUH 2019-09- 031). Participants responded to the questionnaire after giving their consent to participate in the study through written and web-based consent forms. A compensation gift was given to the participants as a reward for their time.

Step 7. Evaluate the Items

(1) Exploratory Factor Analysis Plan

Prior to factor analysis, the mean, standard deviation, skewness, and kurtosis of each item and the correlation coefficients between items were checked through item analysis of the tool. To confirm the suitability of the factor analysis, Bartlett’s sphericity test, was conducted and Kaiser–Meyer–Olkin (KMO) values were checked. Principal component analysis was conducted for factor extraction, and varimax rotation was applied for factor rotation.

To evaluate whether the structure of the factors extracted from the exploratory factor analysis was appropriate, a confirmatory factor analysis was performed. Furthermore, the model fit index of the factor structure was calculated using the maximum likelihood estimation method.

In addition, absolute fit indices, such as the χ^2^ (CMIN) test, root mean square residual (RMR), goodness of fit index (GFI), adjusted goodness of fit index (AGFI), root mean square error of approximation (RMSEA), and standardized root mean square residual (SRMR), and incremental fit indices that are less sensitive to the number of samples, including the normed fit index (NFI), incremental fit index (IFI), Tucker–Lewis index (TLI), and comparative fit index (CFI) were used to evaluate the fitness of the model. A convergent validity test was performed to determine whether the items explained the same concept. In addition, a discriminant validity test was performed to confirm the low correlation between the factors.

(2) Criterion Analysis Plan

A simultaneous validity test was performed for criterion validity, and the Pearson correlation coefficient between Ban’s tool [27] and IHI’s VAP prevention bundle [6] was analyzed.

(3) Reliability Analysis Plan

The reliability of the final tool was verified by calculating Cronbach’s α value (internal consistency reliability) and the Spearman–Brown coefficient and the Guttman half coefficient (split-half reliability).

## 3. Results

### 3.1. Exploratory Factor Analysis

Out of a total of 30 items, 2 items that did not met the normality criteria [37] were removed, resulting in 28 items. The correlation coefficient between the 28 items and the total score ranged from 0.16 to 0.54, and 6 items with correlation coefficients lower than 0.30 were removed [38]. After the removal, Cronbach’s α value for the 22 items was 0.84.

Exploratory factor analysis was performed three times using the principal component analysis method based on varimax rotation to confirm the loading structure and factors of the 22 items selected through item analysis. As a result of the primary factor analysis, all 6 factors with an eigenvalue of 1.0 or higher were extracted [39], and the cumulative explanatory power was 58.5%. After confirming the communality of each item, 4 items that did not satisfy 0.50 or higher were removed [39], and a secondary factor analysis was performed on a total of 18 items.

As a result of secondary factor analysis, 6 factors with an eigenvalue of 1.0 or higher were extracted. The cumulative explanatory power was found to be 64.9% and no items had a factor loading of less than 0.50. However, item 16, “The condensate water accumulated in the ventilator circuit is frequently emptied”, in regard to “ventilator circuit management” was deleted as it was different in content compared to two other items it was grouped with (“Factor 4” item 7, “Oral care is using chlorhexidine (0.12–2%)” and item 8, “Oral care is performed every 4–8 h”), which were about “oral care”.

In addition, among the items grouped with “Factor 3”, “factor loading” of item 12 (0.54) and 13 (0.58) also appeared to be above 0.40 in the factor loading results for “Factor 1” (item 12, 0.50; item 13, 0.46). Because the load of other factors identified to overlap (0.40) could make it difficult to analyze the factor structure [39], a tertiary factor analysis was performed with the number of factors set to 7.

As a result, 7 factors were extracted with 71.8% of the cumulative explanatory power. The factor loading was shown to be 0.57–0.84, meeting all standards and leaving no need to remove items (Table 2).

### 3.2. Confirmation Factor Analysis

Confirmatory factor analysis was performed to test the fit of the theoretical model and determine whether the structure of the seven factors extracted from the exploratory factor analysis was appropriate. The fit index of the model evaluated through confirmatory factor analysis was x^2^ = 137.8 (*p* < 0.001), with an absolute fit index at RMR = 0.01, RMSEA = 0.03, GFI = 0.94, and AGFI = 0.91. This implies that the fitness of the model was good. The multiple fit indices NFI, IFI, TLI, and CFI all exceeded 0.90, which indicates good results; the SRMR value was 0.03, which also indicates good results [40]. As a result of checking the standardized factor loading where each item explains the factor, all the items were between 0.50 and 0.95, meeting the ideal standard of 0.50 or more or 0.95 or less [39,41].

The correlation coefficients between all latent variables were between 0.05 and 0.57, all of which were less than 0.90 (Table 3).

### 3.3. Convergent Validity and Discriminant Validity

As a result of confirmatory factor analysis, all standardized factor loadings were 0.50 or higher and 0.95 or lower. As the results were statistically significant, it could be considered that convergent validity was high. Convergent validity was also confirmed as the average variance extracted (AVE) of the tool in this study was found to be 0.51–0.79, meeting the standard of 0.50 or above. In addition, with construct reliability (CR) at 0.71–0.88, meeting the standard of 0.70 or above, convergent validity was confirmed [39,41] (Table 3).

From the correlation analysis for discriminant validity, all factors were found to be within the recommended level of 0.70. The square root of the average variance extracted (AVE) was 0.72–0.89, and the correlation coefficient of each factor was 0.02–0.57. This implies that the square root of the AVE was larger than the correlation coefficient (φ) of each factor. It appeared that the confidence interval (correlation coefficient ± 2 × standard error) of the correlation coefficient (φ) between factors did not include 1.0, rejecting the hypothesis that each concept was identical and verifying discriminant validity [42]; therefore, it can be concluded that these factors were properly extracted (Table 4).

### 3.4. Criterion Validity Analysis

The Pearson correlation coefficient of 17 items of the VAP preventative behavior measurement tool and 43 items of Ban’s [27] tool was 0.72 (*p* < 0.001), and the figure was 0.78 (*p* < 0.001) with five items of IHI’s [6] VAP bundle, showing a significant positive correlation. The criterion validity was deemed confirmed if the correlation coefficient between two tools is 0.40–0.80; therefore, the criterion validity of the tool was considered attained.

### 3.5. Reliability

The overall reliability coefficient of the final 17 items (Cronbach’s α) was 0.80. For each factor, the “aspiration prevention” of Factor 1 was 0.81; “ventilator management” of Factor 2, 0.80; “spontaneous awakening trials and spontaneous breathing trials” of Factor 3, 0.74; “subglottic suction” of Factor 4, 0.71; “suction system management” of Factor 5, 0.87; “standard precaution” of Factor 6, 0.75; and “oral care” of Factor 7, 0.75. In summary, the reliability of the tool met the standard of > 0.60 [32]. For split-half reliability, the Spearman–Brown and Guttman coefficients were all 0.88. A high correlation coefficient confirmed that each item represents the same concept.

Step 8. Optimize Scale Length

Through the validity and reliability verification process conducted thus far, the number of items in the VAP preventive behavior measurement tool was finally confirmed to range from 30 to 17. The seven factors were as follows: Factor 1 (4 items), “aspiration prevention”; Factor 2 (3 items), “ventilator management”; Factor 3 (2 items), “spontaneous awakening trials and spontaneous breathing trials”; Factor 4 (2 items), “subglottic suction”; Factor 5 (2 items), “suction system management”; Factor 6 (2 items) “standard precaution”; and Factor 7 (2 items), “oral care”. Ranging between 17 and 68 points, a higher sum indicated a high compliance rate of VAP prevention behaviors.

## 4. Discussion

This methodological study identified the components of VAP preventive behaviors targeting ICU nurses and developed a tool to measure the implementation of VAP preventive behaviors. In the construct validity verification stage, the first factor consisted of elements related to aspiration prevention and included endotracheal tube cuff management, which was not included in the existing tools. The pressure of the endotracheal tube cuff should be maintained at 20 cm H_2_O or more, but if the pressure rises excessively, ischemia of the tracheal mucosa occurs and damages the trachea [8]; hence, the pressure was fixed at 20–30 cm H_2_O [35]. The “ventilator management” factor was about the circuit management and humidification that connects the patient and the ventilator. Although not included in the VAP prevention bundle, it was actual preventive behavior implemented in ICUs.

Existing tools in Korea do not mention ventilator circuit replacement [38,40] and measure regular replacement after using the circuit for a certain period of time (7–14 days) [27,29]. This study reflected the latest guidelines related to ventilator management, including the items “The ventilator circuit is not replaced regularly, and it is only replaced when there is visible contamination or functional disorders” [8,34,35] and “The humidifier inside the ventilator circuit uses sterile distilled water”.

The “Spontaneous awakening trials and spontaneous breathing trials” factor is commonly included in guidelines of healthcare institutions or existing tools developed overseas [6,8,33,34,35,36,37]. This is based on the recent finding that sedation interruption and spontaneous breathing trials shorten the period the patient is reliant on a ventilator [43] and increase the success rate of endotracheal tube extubation [44]; however, as no existing tools in Korea include this factor, this is a differentiated study that reflects the latest knowledge. The “subglottic suction” factor is related to the aspiration of oropharyngeal secretions accumulated in the subglottic region above the endotracheal tube cuff. This factor is strongly recommended by PHAC, SHEA [34], and the KDCA [8]; however, during the focus group interview, the respondents revealed that only one existing tool [28] in Korea measured the factor and that they did not know about the necessity of subglottic suction or endotracheal intubation capable of subglottic suction.

This is presumed to result from the limited use of endotracheal intubation capable of subglottic suction because it is not covered by medical insurance in Korea; therefore, even if subglottic suction is impossible under the current domestic medical reality, the measurement tool can be used to recognize that subglottic secretion aspiration is a VAP preventive behavior. The tool also suggests situations where subglottic suction is necessary, which differentiates it from existing tools.

Regarding the “suction system management” factor, there are no differences in VAP prevention between the different types of systems [8]; thus, prevention behaviors to be observed in ICUs in Korea were included in each system where two types (closed and open) of suction systems are used simultaneously.

With regard to the “oral care” factor, the IHI in the United States [6] and the KDCA in Korea [8] both strongly recommend providing oral care using chlorhexidine for patients on mechanical ventilation; however, most existing tools do not include an item on oral care [23,43] nor present oral care solutions [27,28,29]. The Betsy Lehman Center [44] and ICSI [36] recommend that medical personnel carry out oral care every 4 and 6–8 h, respectively. In regard to the daily routine and intervals, existing tools did not provide suggestions on the number of times or intervals for oral care [27,28,29,30,45]. This study considered the current manpower status in ICUs in Korea through interviews with ICU nurses and widened the interval of oral care to 4–8 h. It is necessary to re-examine the frequency of oral care when the manpower problem is resolved in the future.

The last factor, “standard precaution”, is related to the accessories used by patients on mechanical ventilation and those with a tracheostomy tube. While there is no mention of existing tools, this study is meaningful in that it broadened the scope of VAP prevention to include patients with tracheostomy tubes. It also broadened the range of VAP prevention behaviors to include peripheral devices and accessories, such as oxygen masks, tubes, and Ambu bags, used by patients on mechanical ventilation. In particular, it differs in that it is the first study in Korea to include items such as endotracheal tube cuff pressure confirmation and scope of management, spontaneous awakening trials and spontaneous breathing trials, and management by the suction system in the measurement tool.

The results of the correlation analysis between Ban’s ventilator-associated pneumonia management survey [27] and IHI’s VAP bundle [6] conducted to confirm the criterion validity showed a significant positive correlation with both tools. Ban’s tool [27] was the first tool to be developed in Korea, and is still used in nursing research, albeit modified and supplemented. Meanwhile, the VAP bundle of the IHI [6] is currently used as the standard in clinical practice; therefore, it can be said that criterion validity was secured in both the nursing academic field and the tools used in actual clinical practice. This result shows that even though Ban’s tool [27] has 43 items and the tool developed in this study has 17 items (less in number), the measurement effect was approximately the same.

Previous tools [27,28,29,30,45] had only been developed through a literature review without a conceptual identification process through fieldwork in the clinical field; thus, they were limited in that only the content validity test and the reliability test were performed by experts. The tool developed in this study identified the concept through fieldwork and focus group interviews with ICU nurses and infection control unit nurses. Another strength is that the tool was developed through the evaluation stages of content validity, construct validity, discriminant validity, convergent validity, criterion validity, and reliability testing, thereby supplementing methodological limitations.

As the tool developed in this study consisted of seven factors and 17 items, and the survey took less than nine minutes, it was suitable for busy intensive care nurses to complete. It was adequate in terms of the number of items and the required time so that the participants would not give insincere responses or feel fatigued; moreover, the data are reliable because it is a self-evaluation tool. In addition, while it is a method of calculating VAP prevention behavior compliance rates by summing all items, it can be used as a subscale that measures the compliance rate for each factor by summing the scores of the items belonging to each subfactor.

### Limitation and Practical Implications

The following are the limitations of the study and suggestions for future research. First, the participants of this study were nurses from the intensive care units of general hospitals with 800 beds or more only. Tools should be developed after expanding the scope of the study to include participants from hospitals of various sizes. 

Second, the field research in this study involved interviewing ICU nurses; however, future research should develop tools through in-depth interviewing of multidisciplinary personnel related to the treatment of patients on mechanical ventilation.

Finally, the degree of VAP preventive action performance according to career or department of intensive care unit nurses should be measured. This could be used to establish strategies to promote the implementation of differentiated preventive actions in areas with low adherence.

This study is significant in that it developed a tool to measure the implementation of VAP preventive behaviors, and verified its validity, reliability, and adequacy. It is also significant because it has developed the only tool to measure the implementation of VAP preventive behavior that has its validity, reliability, and model adequacy tested in nursing in Korea and abroad.

## 5. Conclusions

This methodological study aimed to develop a tool to measure the implementation of VAP preventive behavior and verify its validity and reliability. The items were selected through literature review, focus group interviews, and preliminary surveys, and the validity and reliability of the selected items were verified through this survey. The VAP preventive behavior implementation measurement tool consisted of 17 items and 7 factors. It was measured on a 4-point scale, and it was interpreted that the VAP preventive behavior compliance rate (Appendix A) increased with the increasing total score. This tool can measure the degree of implementation of VAP precautions in intensive care units and can be used to recognize the importance of and improve the implementation of VAP preventive actions.

## Figures and Tables

**Table 1:** Demographic characteristics of participants.

Characteristics	Categories	Data Set EFA (*n* = 180) *n* (%) or M ± SD	Data Set CFA (*n* = 272) *n* (%) or M ± SD
Gender	Man	22 (12.2)	40 (14.7)
	Woman	158 (87.8)	232 (85.3)
Age (years)		29.3 ± 4.90	28.9 ± 4.80
Level of education	College	19 (10.6)	27 (9.9)
	University	129 (71.7)	191 (70.2)
	≥Graduate	32 (17.8)	54 (19.8)
Current work unit	MICU	58 (32.2)	85 (31.3)
	SICU	68 (37.8)	78 (28.7)
	NCU	16 (8.9)	33 (12.1)
	CICU	3 (1.7)	8 (2.9)
	ECU	32 (17.8)	66 (24.3)
	Others	3 (1.7)	2 (0.7)
Clinical career (years)	≤1	16 (8.9)	25 (9.2)
	1~<5	87 (48.3)	139 (51.1)
	5~<10	45 (25.0)	78 (28.7)
	10~<15	26 (14.4)	22 (8.1)
	≥15	6 (3.3)	8 (2.9)
		5.64 ± 4.33	5.12 ± 4.04
Region of hospital	Seoul	85 (37.2)	152 (55.9)
	Daejeon	48 (22.8)	86 (31.6)
	Gyeongsang-do	9 (5.1)	19 (7.2)
	Gyeonggi-do	7 (3.9)	6 (2.2)
	Chungcheong-do	29 (16.1)	6 (2.2)
	Jeolla-do	2 (1.2)	3 (1.1)
Scale of Hospital (beds)	800~899	124 (68.9)	181 (66.5)
	900~999	15 (8.3)	16 (5.9)
	≥1000	41 (22.8)	75 (27.6)

EFA = Exploratory factor analysis; CFA = Confirmatory factor analysis; M = Mean; SD = Standardized deviation; MICU = Medical intensive care unit; SICU = Surgical intensive care unit; NCU = Neuro (surgical) intensive care unit; CICU = Cardiac intensive care unit; ECU = Emergency intensive care unit.

**Table 2:** Factor loading of items according to the factors and eigenvalue (17 Items) (*n* = 180).

Factors	Item	Item Analysis				Factor Loading						
		M ± SD	ITC	Alpha If Item Deleted	Communality	1	2	3	4	5	6	7
1. Prevention of aspiration	Q2	3.61 ± 0.55	0.42	0.84	0.71	0.76	0.10	0.03	0.22	0.21	−0.11	−0.02
	Q1	3.74 ± 0.47	0.41	0.84	0.72	0.76	0.14	0.17	−0.20	0.03	0.15	0.17
	Q3	3.43 ± 0.81	0.53	0.83	0.73	0.65	0.09	−0.14	0.37	0.20	0.06	0.28
	Q6	3.69 ± 0.61	0.41	0.84	0.76	0.60	0.12	0.42	−0.02	−0.11	0.41	−0.13
2. Ventilator circuit management	Q18	3.60 ± 0.52	0.42	0.84	0.74	0.10	0.81	0.01	0.20	0.03	0.15	0.01
	Q14	3.53 ± 0.73	0.50	0.83	0.74	0.16	0.81	0.19	0.06	0.06	0.03	0.05
	Q15	3.63 ± 0.60	0.34	0.84	0.62	0.06	0.57	−0.19	0.45	0.09	−0.13	0.13
3. Spontaneous awakening trials and spontaneous breathing trials	Q10	2.42 ± 1.05	0.51	0.83	0.79	0.08	0.11	0.82	0.16	0.14	−0.06	0.19
	Q11	2.59 ± 0.98	0.44	0.83	0.74	0.09	−0.03	0.78	0.24	0.06	0.04	0.20
4. Subglottic suction	Q13	3.18 ± 0.82	0.47	0.83	0.70	0.06	0.15	0.25	0.76	−0.01	0.17	−0.01
	Q12	3.43 ± 0.77	0.43	0.83	0.61	0.07	0.25	0.26	0.67	0.01	0.10	−0.01
5. Suction system management	Q22	3.87 ± 0.36	0.30	0.84	0.77	0.09	−0.04	0.08	0.12	0.82	0.18	−0.15
	Q23	3.83 ± 0.48	0.41	0.84	0.74	0.15	0.19	0.09	−0.09	0.79	0.10	0.13
6. Standard precaution	Q28	3.90 ± 0.30	0.36	0.84	0.78	0.07	−0.06	0.05	0.18	0.15	00.84	0.08
	Q30	3.79 ± 0.42	0.39	0.84	0.68	0.06	0.25	−0.08	0.04	0.22	0.67	0.30
7. Oral care	Q8	2.93 ± 0.92	0.39	0.84	0.70	0.20	0.06	0.09	0.14	−0.07	0.15	0.77
	Q7	3.18 ± 0.82	0.31	0.84	0.63	−0.02	0.04	0.27	−0.10	0.04	0.09	0.73
Eigen value						2.12	1.94	1.84	1.70	1.54	1.54	1.50
Explained variance (%)						12.5	11.4	10.8	10.0	9.1	9.0	8.8
Cumulative variance (%)						12.5	23.9	34.7	44.8	53.9	63.0	71.8
KMO (Kaiser–Meyer–Olkin)						0.75						
Bartlett’s Sphere Formation Test						X^2^ = 840.75, df = 136, *p* < 0.000						

M = Mean; SD = Standardized deviation; ITC = Corrected item to total correlation; alpha if item deleted = Cronbach’s alpha if the item was deleted.

**Table 3:** Confirmatory factor analysis findings (*n* = 272).

Fitness Index	$x^{2}$	df	GFI	AGFI	NFI	IFI	TLA	CFI	RMSEA	RMR	SRMR
Criteria			≥0.90	≥0.90	≥0.90	≥0.80	≥0.80	≥0.95	≤0.05	≤0.05	≤0.05
VAP prevention	137.8	98	0.94	0.91	0.92	0.97	0.96	0.97	0.03	0.01	0.03
Factors	Item (17)	Standardized estimate (*β*)		Standard error (SE)		Critical ratio (C.R.)		*ρ*	Error variances	AVE	CR
1. Prevention of aspiration	1	0.74							0.10	0.51	0.81
	2	0.70		0.09		10.33		<0.001	0.13		
	3	0.68		0.10		10.11		<0.001	0.15		
	6	0.75		0.09		10.99		<0.001	0.11		
2. Oral care	7	0.85							0.17	0.61	0.75
	8	0.70		0.13		6.28		<0.001	0.35		
3. Spontaneous awakening trials and spontaneous	10	0.70							0.52	0.60	0.75
Breathing trials	11	0.84		0.13		8.47		<0.001	0.26		
4. Subglottic suction	12	0.71							0.25	0.55	0.75
	13	0.77		0.15		8.01		<0.001	0.26		
5. Ventilator circuit management	14	0.94							0.01	0.65	0.84
	15	0.50		0.06		8.66		<0.001	0.26		
	18	0.85		0.05		15.89		<0.001	0.07		
6. Suction system management	22	0.95							0.01	0.79	0.88
	23	0.82		0.08		12.17		<0.001	0.07		
7. Standard precaution	28	0.84							0.04	0.60	0.75
	30	0.71		0.09		9.37		<0.001	0.08		

Df = Degree of freedom; GFI = Goodness of fit index; AGFI = Adjusted goodness of fit index; NFI = Normed fit index; IFI = Incremental fit index; TLI = Tucker–Lewis index; CFI = Comparative fit index; RMSEA = Root mean square error of approximation; RMR = Root mean square residual; SRMR = Standardized root mean square residual; AVE = Average variance extracted; CR = Composite reliability.

**Table 4:** Convergent and discriminant validity tests.

	Factors						
	1	2	3	4	5	6	7
1. Prevention of aspiration	0.72 †						
2. Oral care	0.25	0.78 †					
3. Spontaneous awakening trials and spontaneous breathing trials	0.34	0.43	0.77 †				
4. Subglottic suction	0.29	0.22	0.52	0.75 †			
5. Ventilator management	0.28	0.13	0.07	0.394	0.80 †		
6. Suction system management	0.42	0.05	0.02	0.17	0.17	0.89 †	
7. Standard precaution	0.44	0.24	0.04	0.35	0.33	0.57	0.77 †
Factor A ↔ Factor B	Φ	SE		Φ − 2 × SE		Φ + 2 × SE	
PA ↔ OC	0.25	0.02		0.21		0.29	
PA ↔ SAT & SBT	0.34	0.02		0.29		0.39	
PA ↔ SS	0.29	0.02		0.25		0.33	
PA ↔ VM	0.28	0.01		0.26		0.30	
PA ↔ SM	0.42	0.01		0.40		0.45	
PA ↔ SP	0.44	0.01		0.42		0.46	
OC ↔ SAT & SBT	0.43	0.04		0.33		0.52	
OC ↔ SS	0.22	0.03		0.15		0.30	
OC ↔ VM	0.13	0.01		0.10		0.16	
OC ↔ SM	0.05	0.01		0.01		0.08	
OC ↔ SP	0.24	0.01		0.21		0.28	
SAT & SBT ↔ SS	0.52	0.04		0.42		0.62	
SAT & SBT ↔ VM	0.07	0.01		0.03		0.10	
SAT & SBT ↔ SM	0.02	0.02		0.00		0.07	
SAT & SBT ↔ SP	0.04	0.01		0.00		0.08	
SS ↔ VM	0.39	0.01		0.36		0.42	
SS ↔ SM	0.17	0.01		0.13		0.20	
SS ↔ SP	0.35	0.01		0.31		0.38	
VM ↔ SM	0.17	0.01		0.15		0.19	
VM ↔ SP	0.33	0.01		0.32		0.35	
SM ↔ SP	0.57	0.01		0.55		0.60	
Criteria	whether (Φ ± 2 × SE) includes 1.0						

† Square root of AVE; Φ = Correlation; PA = Prevention of aspiration; OC = Oral care; SAT = Spontaneous awakening trials; SBT = Spontaneous breathing trials; SS = Subglottic suction; VM = Ventilator circuit management; SM = Suction system management; SP = Standard distribution.

## Data Availability

Data cannot be shared publicly because of restrictions by the Konyang University Hospital Institutional Review Board. Data are available from the Konyang University Hospital Institutional Data Access/Ethics Committee for researchers who meet the criteria for access to confidential data. Data requests can be addressed to the Konyang University Hospital Institutional Review Board (82-42-600-9057, leesh@kyuh.ac.kr).

## Appendix A. VAP Preventative Behavior Measurement Tool

**Table:**

No	Item	I Never Do It	I Seldom Do It	I Mostly Do It	I Always Do It
1	Except for contraindicated patients, elevate at an angle of 30–45 degrees the head of the bed	①	②	③	④
2	Cuff pressure should be maintained at 20–30 cm H_2_O	①	②	③	④
3	Regularly check and control of tracheal cuff pressure using a manual manometer	①	②	③	④
4	Regularly check the proper positioning of the nasogastric tube	①	②	③	④
5	Oral care is performed with chlorhexidine (0.12% or 2%)	①	②	③	④
6	Oral care is performed every 4–8 h	①	②	③	④
7	Perform subglottic secretion suction (subglottic secretion: oropharyngeal secretions in the subglottic region above the cuff of the endotracheal tube)	①	②	③	④
8	Before deflating the cuff of an endotracheal tube, before moving the tube, or patient position change ensure that secretions are cleared from above the tube cuff	①	②	③	④
9	Except for contraindicated patients, Daily Perform spontaneous awakening trials with sedatives turned off	①	②	③	④
10	Daily perform spontaneous breathing trials once a day	①	②	③	④
11	Change the ventilator circuit when it is visibly soiled or mechanically malfunctioning	①	②	③	④
12	Do not flush condensate water from the ventilator circuit into the machine or onto the patient	①	②	③	④
13	Use sterile water to fill ventilator humidifiers	①	②	③	④
14	In patients using an open suction system, use a sterile disposable suction catheter and sterile water for each suction.	①	②	③	④
15	In patients using an closed suction system, a closed suction catheter should be used for each new patient	①	②	③	④
16	Observe aseptic technique when managing the tracheostomy site and replacing the tracheostomy tube	①	②	③	④
17	Do not use the oxygen tube, mask, and ambu bag used for one patient on another patient.	①	②	③	④
